# The Significance of MicroRNAs in the Molecular Pathology of Brain Metastases

**DOI:** 10.3390/cancers14143386

**Published:** 2022-07-12

**Authors:** Frantisek Siegl, Marek Vecera, Ivana Roskova, Martin Smrcka, Radim Jancalek, Tomas Kazda, Ondrej Slaby, Jiri Sana

**Affiliations:** 1 Central European Institute of Technology, Masaryk University, 625 00 Brno, Czech Republic; 451857@mail.muni.cz (F.S.); marek.vecera@ceitec.muni.cz (M.V.); oslaby@med.muni.cz (O.S.); 2 Department of Neurosurgery, University Hospital Brno and Faculty of Medicine of Masaryk University, 625 00 Brno, Czech Republic; roskova.ivana@fnbrno.cz (I.R.); smrcka.martin@fnbrno.cz (M.S.); 3 Department of Neurosurgery, St. Annes University Hospital Brno and Faculty of Medicine of Masaryk University, 656 91 Brno, Czech Republic; radim.jancalek@fnusa.cz; 4 Department of Radiation Oncology, Masaryk Memorial Cancer Institute and Faculty of Medicine of Masaryk University, 656 53 Brno, Czech Republic; tomas.kazda@mou.cz; 5 Department of Biology, Faculty of Medicine, Masaryk University, 625 00 Brno, Czech Republic; 6 Department of Comprehensive Cancer Care, Masaryk Memorial Cancer Institute and Faculty of Medicine of Masaryk University, 656 53 Brno, Czech Republic; 7 Department of Pathology, University Hospital Brno, 625 00 Brno, Czech Republic

**Keywords:** brain metastases, microRNA, metastatic cascade, biomarkers

## Abstract

**Simple Summary:**

Brain metastases are increasingly common in cancer patients and hurt survival and quality of life. Therefore, efforts are increasingly devoted to research into brain metastases and discovering new diagnostic approaches and therapeutic targets. MicroRNAs, which are involved in regulating most cellular processes, including metastasis, appear to be promising molecules in this regard. The number of studies on microRNAs is constantly increasing. This review aims to summarize the current knowledge on the importance of microRNAs in the pathobiology of brain metastases and to suggest possibilities for their use in diagnostic and therapeutic practice.

**Abstract:**

Brain metastases are the most frequent intracranial tumors in adults and the cause of death in almost one-fourth of cases. The incidence of brain metastases is steadily increasing. The main reason for this increase could be the introduction of new and more efficient therapeutic strategies that lead to longer survival but, at the same time, cause a higher risk of brain parenchyma infiltration. In addition, the advances in imaging methodology, which provide earlier identification of brain metastases, may also be a reason for the higher recorded number of patients with these tumors. Metastasis is a complex biological process that is still largely unexplored, influenced by many factors and involving many molecules. A deeper understanding of the process will allow the discovery of more effective diagnostic and therapeutic approaches that could improve the quality and length of patient survival. Recent studies have shown that microRNAs (miRNAs) are essential molecules that are involved in specific steps of the metastatic cascade. MiRNAs are endogenously expressed small non-coding RNAs that act as post-transcriptional regulators of gene expression and thus regulate most cellular processes. The dysregulation of these molecules has been implicated in many cancers, including brain metastases. Therefore, miRNAs represent promising diagnostic molecules and therapeutic targets in brain metastases. This review summarizes the current knowledge on the importance of miRNAs in brain metastasis, focusing on their involvement in the metastatic cascade and their potential clinical implications.

## 1. Introduction

The metastatic stages of cancer, involving the systemic spread of cancer cells and the development of secondary tumor foci, are among the most demanding challenges of modern medicine. Brain metastases (BMs) are among the most destructive of these tumor foci, crucially influencing morbidity, survival, and quality of life [1]. It is estimated that 9% to 17% of tumors establish distant metastases in the brain; however, the incidence of BMs increases slightly every year [1,2]. The main reason for this increase could be the introduction of new and more efficient therapeutic strategies that lead to longer survival but, at the same time, cause a higher risk of brain parenchyma infiltration. In addition, the advances in imaging methodology, which provides earlier identification of BMs, may also be a reason for the higher recorded number of patients with BMs [3,4]. Despite the improvements in diagnostics and patient management, and the development of new therapeutics, BMs are commonly a fatal event in cancer progression, with patient survival usually being less than 10 months from the diagnosis of metastatic cancer [5].

Tumors with the highest metastatic tendencies toward the brain are lung carcinomas (16.3 to 36%), breast carcinomas (5 to 30%), melanomas (6 to 11%), renal cell carcinomas (RCC) (2 to 16%), and colorectal carcinomas (CRC) (1 to 4%) [6,7,8]. Significantly less common are BMs progressing from oesophageal tumors, bladder cancer, prostate cancer, testicular cancer, ovarian cancer, endometrial cancer, or choriocarcinoma [8]. In the context of lung cancer, patients with adenocarcinoma are more likely to be burdened by the onset of BMs (26.8%), followed by patients with non-small cell lung cancer (NSCLC) (25.6%), small cell lung carcinoma (23.5%), squamous lung carcinoma (15.9%), and bronchioloalveolar carcinoma (15.5%) [9]. For patients suffering from breast cancer, the highest risk of the development of BMs is correlated with human epidermal growth factor receptor 2 positive (HER-2+) breast cancer, with up to half of patients developing BMs during the course of the disease [10,11].

Different organ tropism could be partially explained by the “seed and soil” theory, which compares the metastatic cell with the seed, with the highest chance to survive in the most favorable microenvironment (soil) of specific distant organs [12]. Furthermore, cells themselves could influence the preparation of the most suitable microenvironment via molecules that are exported by exosomes or through the transport of parts of the original microenvironment (for example, in the form of activated fibroblasts) [13,14,15]. A predisposition to organ tropism could also be found in specific cell surface markers, such as cluster of differentiation 44 positive (CD44+) breast cancer cells metastasizing to bone marrow [16]. The identification of such markers and a significantly better understanding of the key signaling pathways involved in the metastatic cascade, as well as increased knowledge about the various levels of regulation of the metastatic cascade, could greatly improve our understanding of the disease, leading to new treatment strategies and therapeutics. 

One such group of molecules could be microRNAs (miRNAs), which are small non-coding RNAs that are responsible for the regulation of gene expression. This review deals with the regulation of miRNAs in metastasis development, including a narrower focus on the roles of miRNAs in the development of brain metastases.

## 2. The Metastatic Cascade and the Development of Metastases

The formation of distant metastases is one of the later stages in cancer development; however, crucial changes in cell biology could occur relatively early in the course of the disease. The so-called metastatic cascade is a process through which the cell phenotype changes, leading to the gain of new properties, especially those properties that allow cells to migrate and colonize surrounding tissue and, subsequently, distant tissue [17]. To invade surrounding tissue, cells undergo epithelial-mesenchymal transition (EMT), in which cell motility and the secretion of microenvironment modulating molecules are promoted, leading to invasion and, ultimately, to intravasation [17,18,19,20]. The critical step during EMT is the induction of resistance to anoikis after the loss of contact with the extracellular matrix (ECM), which is prevented by defects in the death receptor pathway [21]. Cells that change their phenotype due to EMT can enter circulation through the lymphatic or blood paths and spread throughout the organism. At the end of the cascade, extravasation of circulating cancer cells occurs in the capillaries of distant organs, with most cells dying. However, if cells are able to survive in a distant organ microenvironment, they act as the cornerstone for the development of the secondary lesion [22,23].

For the successful formation of the secondary foci and the correct course of the metastatic cascade, specific alterations in cell signaling pathways and metabolism must occur. Key regulators of the metastatic cascade are the specific transcription factors (TFs), which are responsible for the expression of specific molecules, leading to the acquisition of the metastatic phenotype. The well-described TFs involved in EMT are snail family transcriptional repressor 2 (SNAIL2), zinc finger E-box-binding homeobox 1 (ZEB1), ZEB2, twist family BHLH transcription factor 1 (TWIST1), TWIST2, and paired related homeobox 1 (PRRX1) [24,25,26,27,28]. Expression of these TFs is triggered by EMT inductors, which are signals that promote EMT and metastatic development. Among these inductors are molecules that are involved in the signaling pathways of Notch, Wnt, or transforming growth factor β (TGF-β), molecules related to extracellular acidosis caused by hypoxia, or tumor metabolic adaptations leading to higher lactate concentration [29,30,31,32,33,34,35]. Most epithelial tumors arise as non-invasive lesions; however, during their development, they acquire the necessary abilities to spread and form distant metastases due to the influence of EMT-related factors [36]. EMT leads to cytoskeletal reorganization, acquisition of the mesenchymal phenotype, disruption of the basal membrane due to the expression of specific proteases, and the degradation of the ECM [37,38,39,40,41].

After the necessary phenotype changes caused by EMT, cells are able to undergo the process of intravasation [40]. During intravasation, cancer cells must adhere to endothelial cells. This interaction is mediated through receptors and ligands of the Notch pathway [41,42,43]. Endothelial cells subsequently promote cytoskeletal and membrane remodeling and the generation of pore-like structures in the endothelium, allowing cancer cells to enter circulation [44,45]. Subsequent survival during circulation presents another great challenge, mainly due to immune surveillance and pernicious hemodynamic forces [46,47,48]. However, circulating cancer cells develop mechanisms to escape immune surveillance, such as the expression of inhibitors of NK receptors or cooperation with platelets [47,49]. Resistance to the hemodynamic forces results from the mechanical adaptation of cells, which leads to greater stiffness and protection from the plasma membrane damage caused by the circulation of mechanical stresses [48]. In the early stages of EMT, cancer cells start to adapt target organs by creating exosomes carrying molecules that can alter the microenvironment of the target and create a premetastatic niche [15]. Cells that subsequently enter the premetastatic niche have a higher chance of successfully developing into secondary tumors.

The process of extravasation begins with the arrival of migrating cells and their capture in the capillaries due to slower flow, allowing interactions with the endothelium to be established. This leads to rolling on the endothelium and ultimately to extravasation [50,51]. Extravasated cells adjust to the microenvironment, benefiting from perivascular localization, leading to the spread of cancer cells and the growth of secondary tumors [37,51].

### Specifics of the Development of Brain Metastases

Migration toward the brain is supported by the formation of a premetastatic niche, which enables easier cell survival and the establishment of secondary foci. Formation of the premetastatic niche could be achieved by influencing the original microenvironment by the molecules secreted in the form of cancer cells’ extracellular vesicles, or by the accumulation of cells that are responsible for the expression of pro-inflammatory cytokines, which are later responsible for the guidance of circulating cancer cells [15,52]. However, extravasation into the brain is more complicated, in comparison with extravasation into other organs, due to the blood-brain barrier (BBB) that prevents free molecular transport to the interstitial fluid of the brain. The BBB consists of microvascular endothelial cells (BMEC); therefore, to develop BMs, it is essential to disrupt the BBB [53]. In the process of extravasation, adhesive molecules, such as E-selectin, vascular cell adhesion molecule 1 (VCAM-1), intercellular adhesion molecule 1 (ICAM-1), or very late antigen-4 (VLA-4), are expressed to ensure interactions between cancer cells and the BBB. Subsequently, the expression of metalloproteases leads to disruption of the BBB and the entry of cancer cells into the brain [54,55].

After extravasation, re-epithelization is required. Thus, the reverse process to that of EMT, called mesenchymal-epithelial transition (MET), occurs. During this process, the re-expression of epithelial markers, such as E-cadherin, is established, and the expression of mesenchymal markers is restrained [56,57]. Some extravasated cancer cells can even undergo partial MET to maintain a more aggressive phenotype to further spread in the brain [58]. After penetration into the brain, cancer cells are surrounded by reactive astrocytes, the first line of defense of the central nervous system (CNS) [59,60]. Reactive astrocytes immensely reduce the number of cancer cells that are able to form BMs, and the efficacy of creating BMs is rather low. However, specific cells can evade this surveillance and survive [51]. In the brain parenchyma, cancer cells use the support of glial cells, helping them to form and develop secondary loci to a greater extent [61]. Astrocytes could be used for the activation of Notch signaling, enabling more effective colonization [62]. Furthermore, interactions with microglia, leading to aberrant activation of cytokine expression, provide another level of microenvironment adaptation for a further stimulation of growth of BMs [63]. However, only a few molecules were described as direct effectors of interactions between the microenvironment and metastasizing cells in the brain. For example, the plasminogen activator (PA) is responsible for plasmin activation or mobilization of the Fas ligand to kill extravasated cells, although metastasizing cells are capable of producing molecules, such as neuroserpin and serpin B2, that act against PA and thus evade Fas ligand-guided apoptosis [51]. Another protein related to the interaction between metastasizing cells and the microenvironment is melanotransferrin. Its binding to membrane or the presence of soluble protein regulates the capacity of metastasizing cells to migrate, due to the regulation of plasminogen activation [64,65]. Other molecules are responsible for the adjustment of the microenvironment, survival, chemoresistance, or activation of crucial pathways that are responsible for the growth of BMs [66,67].

Cells not only adjust the microenvironment for their needs, they also undergo various changes to benefit from the new surroundings. Breast cancer cells are capable of expressing γ-aminobutyric acid (GABA) receptors and transporters or glutamate decarboxylase for the synthesis of GABA from glutamate, all leading to higher energy gains from the neurotransmitters that are present in the brain [68]. Changes in the expression profiles also occur at the miRNA level. MiRNAs are highly potent gene expression regulators that influence the majority of biological processes, including metastasizing. The dysregulation of the expression of specific miRNAs is indispensable for a successful metastatic cascade and could provide an interesting tool for the diagnostics of metastasis development [69].

## 3. MicroRNAs

In addition to protein-coding RNAs, there are other RNA molecules with various regulatory functions. These are non-coding (ncRNAs) that are not translated into proteins. One large group comprises the short ncRNAs. That group is further subdivided into several subgroups, such as miRNAs, small interfering RNAs (siRNA), and PIWI-interacting RNAs (piRNA) [70,71]. MiRNAs are responsible for the regulation of the gene expression of a wide range of protein-coding genes. They have a huge impact on cellular biology. Moreover, it was found that the dysregulation of various short non-coding molecules is often connected with human pathologies, including cancer [72,73].

MiRNAs are single-stranded RNAs with an approximate length of 22 nucleotides that are encoded in the genome, transcribed into primary miRNA, and finally generated from hairpin-like precursors. Mature miRNAs are incorporated into the ribonucleoprotein complex, which is known as the RNA-induced silencing complex (RISC) [74,75]. The RISC searches for mRNA sequences that are complementary to RISC-associated miRNAs. In the case of a perfect pairing between a miRNA and its target, mRNA degradation occurs [76,77]. In the event of non-perfect pairing, translation is stopped, which is often followed by the degradation of the mRNA [78]. Canonical interactions between mRNA and miRNA occur in the 3′ untranslated region (3′UTR), which is responsible for mRNA stability and translation efficiency [79,80].

Single miRNA may regulate the expression of a wide variety of mRNA molecules. Therefore, miRNAs are indispensable regulators of various biological processes, including the regulation of cell cycles, apoptosis, differentiation, stress responses, and other processes. Their role is crucial at supracellular levels, as they contribute to organ development, homeostasis, and immune response. Additionally, redundancy is the typical characteristic of the miRNA pathway. For example, the deletion of 83% of miRNAs in *C. elegans* did not cause essential dysfunction in development or viability; just 10% miRNA was sufficient to maintain the normal phenotype of the organism [81]. However, Dicer knockout causing complete miRNA depletion led to lethality in the early development stage, suggesting the indispensability of miRNA machinery for embryonal development [82]. 

There is also high miRNA redundancy in higher organisms. For example, the knockouts of various miRNAs were observed to have no phenotypic effect in mice [83]. However, a few miRNAs with crucial and irreplaceable roles during development were identified [84,85]. Various tasks in different biological processes, as well as their dysregulation function in the context of cancer, suggest that miRNAs could be high-impact molecules in processes connected with the metastatic cascade.

## 4. MicroRNAs Involved in the Metastatic Cascade

As indicated, EMT is an important process associated with metastasis. Among the molecules that regulate EMT, miRNAs are known to be highly potent gene expression regulators. Therefore, it is no surprise that many miRNA molecules were described in the context of the specific steps of the metastatic cascade. Molecules with proven biological effects on metastases and at least a partially explored mechanism of action are discussed in this section.

Many molecules, including miRNAs, are altered in tumors, due to various genetic abnormalities, such as chromosomal deletions, amplifications, translocations, or mutations. Furthermore, transcriptional activation or repression, as well as epigenetic changes and miRNA biogenesis defects, play important roles in the dysregulation of miRNA levels. Dysregulated miRNAs are often found in loci that are susceptible to genetic alterations [86]. Those alterations could be drivers that cause specific tumors to have a high metastatic potential. The first steps toward metastasis occur during the early stages of cancer development, which allow cells to acquire important qualities that result in a more malignant phenotype, leading to migration and invasion of surrounding tissue [87]. Phenotypic changes are accompanied by changes in expression patterns. Therefore, the recognition of pre-metastatic stages or early metastatic stages of the disease, for example, through altered miRNA expression profiles, could be highly beneficial.. Moreover, micrometastases or circulating cancer cells are not identifiable by standard procedures. Early capture of these events could have a significant positive impact on the management of patients with metastases [88,89]. MiRNAs can potentially provide potent biomarkers for various applications, including diagnostics, and they are also potential therapeutic targets.

### 4.1. MicroRNAs with a Suppresive Function for Metastases Development

The first study on miRNAs in metastases, by Li Ma et al., was published in 2007. In that study, the miRNA expression of metastasizing breast carcinoma cancer cells was compared with that of normal epithelial breast tissue. Several metastasis-associated miRNAs were identified; among them, miR-10b was reported as the key molecule in the development of metastases in xenograft models. The overexpression of miR-10b led to greater motility and invasiveness of cancer cell lines via the inhibition of metastasis suppressors, such as homeobox D10 (HOXD10), neurofibromin 1 (NF1), Krüppel-like factor 4 (KLF4), or phosphatase and tensin homolog (PTEN), while the miR-10b silencing led to lower tumor growth in vivo and less invasiveness in vitro [90].

The members of the miR-200 family, which are important metastasis regulators, are responsible for the suppression of the EMT regulators ZEB1 and ZEB2 [91]. ZEBs, on the other hand, can control the expression of the miR-200 family by binding to the specific promoter regulation sequences of miR-200 genes. This negative feedback loop is a possible major regulatory system of EMT [92]. Together with the miR-200 family, the cluster miR-183~96~182 was described as an important player in the inhibition of metastasis development. In lung cancer, miRNAs of this cluster, together with the miR-200 family, inhibit forkhead box F2 (FOXF2), an inhibitor of E-cadherin [93]. A similar regulation loop to that of miR-200-ZEB1/2 is found in breast cancer, where miR-203 reduces the expression of Snail2, another important inductor of EMT [94]. Interestingly, miR-200a is associated with prometastatic abilities, such as resistance to anoikis in breast cancer. MiR-200a silences yes-associated protein 1 (YAP-1), as well as other proapoptotic genes, such as phorbol-12-myristate-13-acetate-induced protein 1 (PMAIP1/NOXA), B-cell lymphoma 2 (Bcl-2) associated X protein (BAX) or Bcl-2-like protein 1 (BCL2L11/BIM), a process that correlates with the higher metastatic potential in breast cancer cells with upregulation of this specific miRNA [95].

MiR-142-3p, another miRNA associated with the metastatic stages of breast cancer, targets BTB domain and CNC homolog 1 (BACH-1). BACH-1 regulates the migration and invasiveness of breast carcinoma cells. By upregulating miR-142-3p, the metastatic potential of breast cancer cells is reduced due to the inhibition of other important molecules for metastasizing, such as C-X-C chemokine receptor type 4 (CXCR4), matrix metalloproteinase-9 (MMP9), or vascular endothelial growth factor receptor (VEGFR), and the expression of protective miRNAs, such as miR-330, miR-145, and miR-34a, is initiated [96]. MiR-34a is one of the key regulators of EMT. In hypoxic conditions, hypoxia-inducible factor 1α (HIF-1α) represses the expression of miR-34a in tumor protein 53 (TP53)-mutated CRC cells, which leads to hypoxia-induced EMT through activation of signal transducer and activator of transcription 3 (STAT3). Therefore, the silencing of miR-34a and STAT3 activation is one of the initial crucial steps in the activation of EMT [97].

### 4.2. Metastases Promoting microRNAs

Among oncogenic miRNAs, synergistic effects are often seen, as in the case of molecules miR-199a-3p, miR-199a-5p, and miR-1908 in melanoma. These miRNAs target the DnaJ heat shock protein family (HSP40) member A4 (DNAJA4) and the Apolipoprotein E (APOE). DNAJA4 inhibits metastases through the upregulation of APOE, which is the central molecule of this pathway. APOE is secreted by melanoma cells and targets the low density lipoprotein receptor-related protein 1 (LRP1) receptor on other melanoma cells and the LRP8 receptor on endothelial cells, leading to the inhibition of migration of melanoma cells [98]. In melanoma, another miRNA molecule, miR-214, is prometastatic. MiR-214 silences cell adhesion molecule 1 (CADM1), a known tumor suppressor, thus leading to higher migration and invasion of melanoma cells and EMT promotion, in which miR-214 contributes to annoikis resistance and subsequently to extravasation [99,100]. The relevance of the miR-199/miR-214 cluster was also described in triple negative breast carcinoma (TNBC); however, in TNBC, the overexpression of miRNAs from the miR-199/miR-214 cluster leads to the inhibition of EMT, higher expression of epithelial markers E-cadherin and β-catenin, and decreased expression of the mesenchymal marker SNAIL2 (also known as SLUG) [101]. MiR-212-5p is another molecule that is connected to TNBC. The upregulation of miR-212-5p leads to the inhibition of invasiveness of cancer cells in vitro and lower tumorigenicity and metastasis formation in vivo. By upregulating miR-212-5p, EMT is suppressed, leading to higher expression of E-cadherin and, in contrast, lower expression of vimentin. The molecular target for miR-212-5p is PRRX2, a transcription coactivator induced by TGF-β [102]. Furthermore, miR-19b is related to the higher metastatic potential of TNBC, as it lowers the levels of myosin regulatory light chain interacting protein (MYLIP), the protein of the ezrin, radixin, and moesin (ERM) family. Upregulation of miR-19b is also connected with the downregulation of E-cadherin and the higher expression of ICAM-1 and Integrin β1. The higher migration rate and invasiveness is also connected with the acquired phenotype of cells with upregulated miR-19b [103].

During intravasation, proteases play a crucial role in the destruction of cell junctions and in the escape of metastasizing cells into circulation. Their expression is also regulated by miRNAs. MiR-1258 targets heparanase, an endoglycosidase that cleaves heparan sulphate, releasing growth and heparin-bonded angiogenic factors that are stored in ECM. MiR-1258 downregulates heparanase, leading to the disruption of the heparanase pathway, lower phosphorylation of Akt and epidermal growth factor receptor (EGFR), and the downregulation of cyclooxygenase-2 (COX2) and MMP9, which are responsible for the destruction of ECM and cell junctions [104]. In addition, miR-139-5p causes the downregulation of MMPs, particularly MMP7 and MMP9, by inhibiting Notch signalization [105]. MMP9 expression is also reduced by miR-194 and the downregulation of the ERK-MMP9 pathway [106]. Another metalloprotease MMP2 is upregulated by miR-194, which supports the colonization of distant organs by circulating cancer cells [107]. Other miRNAs are connected to EMT indirectly. For example, the miR-103/107 family attenuates miRNA biogenesis by targeting Dicer, leading to the downregulation of the important protective miR-200 family. Downregulation of miR-200 causes the induction of EMT by the transcription factors ZEB1 and ZEB2, leading to the dissemination of breast cancer cells [108].

### 4.3. MicroRNAs Involved in the Premetastaci Niche Formation and Microenvironment Modulation

Extracellular microvesicles could also be involved in the metastatic cascade, as they play a crucial role in the formation of a premetastatic niche. MiR-21 is secreted to the liver, where it is recognized by macrophages and stimulates their polarization and interleukin-6 (IL-6) production. Secreted IL-6 upregulates miR-21 recruitment, leading to inflammation and the formation of a premetastatic niche that attracts and supports the mestastisizing of CRC cells into the liver [109]. In addition, other miRNAs, including miR-25-3p, miR-130b-3p, or miR-425-5p, were observed to be secreted to the liver to prepare the premetastatic niche for CRC cells in the same way as miR-21 [110]. Furthermore, exosomes produced by cells other than cancer cells could be highly potent in the promotion of metastasis. For example, miR-223, contained in microvesicles produced by activated macrophages, is responsible for a more aggressive phenotype of recipient cells [111].

Another level of regulation of metastasis development through microvesicles secretion was described by Zhou et al. [112]. In that study, maturated miRNAs released in exosomes were received by endothelial cells. One of these miRNAs was miR-105, which is responsible for the downregulation of tight junction protein 1/zonula occludens-1 (ZO-1) protein, a crucial component of tight junctions in endothelial cells. Together with the internalization of occludin, higher levels of miR-105 led to endothelial barrier destruction, causing enormous facilitation of extravasation for cells secreting exosomes containing miR-105 in vitro. In addition, the presence of miR-105-containing exosomes led to higher number of metastases in vivo. Although miR-105 was not responsible for alterations in cell proliferation, it enabled easier penetration through the endothelial barrier of distant organs. Interestingly, the miR-105 levels in the serum of patients were correlated with the development of distant metastases [112]. 

MiRNAs, which are dysregulated in biofluids, are at the peak of scientific interest in applied cancer research, as they are believed to be a promising tool for early revelation of metastasis development and subsequent changes in patient therapy, possibly leading to prolonged survival. Therefore, the elucidated role of miRNAs, such as miR-105, in metastasis formation supports their potential use as potent biomarkers.

**Figure 1:**
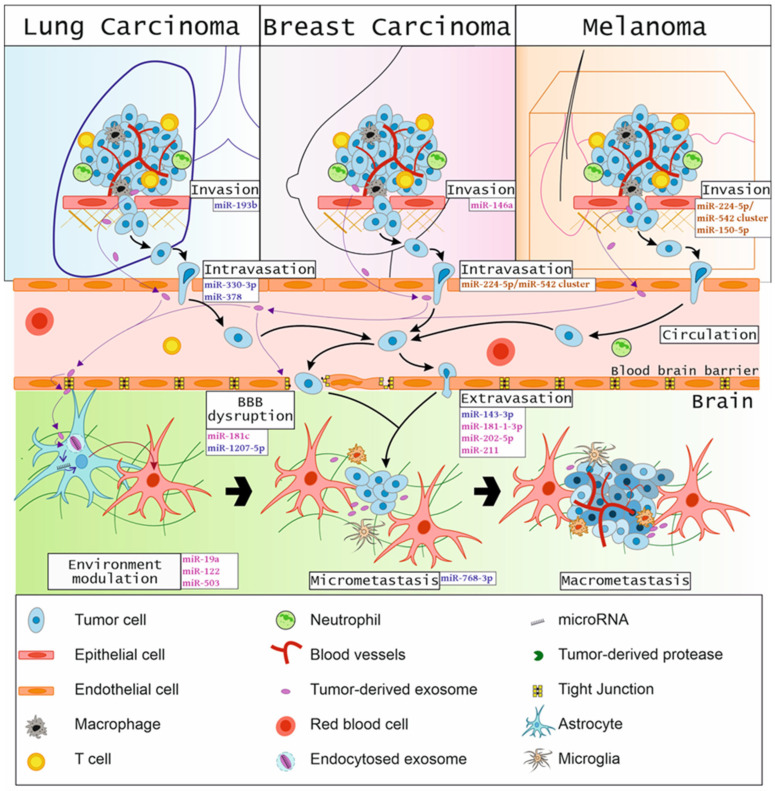
Overview of the involvement of specific miRNAs in a metastatic cascade toward the brain.

The miRNAs involved in different stages of the brain metastasis cascade are shown in Figure 1.

## 5. MicroRNAs Involved in Brain Cancer Metastases

Given the evidence in the previous section, in the last two decades, many miRNAs have been described in the context of specific steps of the metastatic cascade or associated with the overall metastatic stage of disease. However, studies focused on the role of miRNAs in BMs are rare and mostly performed in only five major groups of tumors that metastasize into the brain: lung carcinoma, breast carcinoma, melanoma, RCC, and CRC.

### 5.1. MicroRNAs in Lung Cancer Brain Metastases

For both men and women, lung carcinoma is the second most common cause of death in the context of cancer. It is the most common cause of cancer-related deaths in general [113,114]. The most important complications for lung carcinoma patients are metastases, as up to 45% of all lung carcinomas metastasize into the brain. Lung carcinomas are the most common tumors that form BMs [115,116,117]. Among the miRNAs mentioned in Section 4, only miR-21 was observed to affect the development of BMs in patients with lung cancer, and its lower expression was correlated with better patient prognosis [118]. MiR-21 was also detected as upregulated in patients with BMs that originated from NSCLC, suggesting its role as a potential biomarker for the development of BMs in NSCLC [119]. Furthermore, the miR-21 promoter was described as a target for STAT3, another important molecule in cancer. STAT3 is capable of modulating miR-21 expression, and the overexpression of miR-21 offsets the effects of STAT3 knockdown, such as reduced proliferation, self-renewal, or migration, suggesting the importance that the STAT3/miR-21 pathway presents in BMs of lung cancer [120].

#### 5.1.1. MicroRNAs with a Suppresive Function for Metastases Development in Lung Cancer

Among miRNAs with suppressive effects in the development of lung carcinoma BMs is miR-768-3p, which is significantly reduced in lung carcinoma cells that are co-cultured with astrocytes. MiR-768-3p targets K-Ras, the regulator of cell viability and a promoter of chemoresistance. The cause of miR-768-3p downregulation is probably found in the brain microenvironment, as its expression is higher in primary tumors [121]. In addition, miR-193b downregulation occurs in BMs of lung cancer. MiR-193b is a dual-strand tumor suppressor, as restoration of its expression from both strands leads to a decrease in the metastatic potential of cells, due to the inability of the cells to invade and migrate. Both miRNAs (miR-193b-3p and miR-193b-5p) target cyclin D1 (CCND1), Ajuba, and heart development protein with EGF like domains 1 (HEG1), and the silencing of their expression acts against BM formation [122]. The mechanism of miRNA action is also affected by other molecules, as in the case of miR-215-3p, which is downregulated in lung carcinomas that metastasize into the brain due to the sponging activity of lncRNA lnc-REG3G-3-1. Downregulation of miR-215-3p leads to higher cell viability, migration, invasiveness, and expression of leptin and solute carrier family 2 member 5 (SLC2A5), followed by significant upregulation of VEGF, serine/threonine/tyrosine interacting like 1 (STYXL1), and flavin adenine dinucleotide synthetase 1 (FLAD1) mRNA levels and downregulation of Akt, phosphoinositide 3-kinase (PI3K), and sex-determining region-box transcription factor 4 (SOX4) mRNA levels. Higher leptin expression plays a crucial role in the promotion of metastases, especially due to the activation of the mammalian target of rapamycin (mTOR) pathway [123].

Another miRNA that acts as a tumor suppressor in lung carcinoma BMs is miR-217, which targets sirtuin 1 (SIRT1), an nicotinamide adenine dinucleotide (NAD)-dependent deacetylase that inhibits tumor protein p53. Higher levels of miR-217 lead to lower migration and viability of lung carcinoma cells and lower expression of MMP9 [124]. Furthermore, miR-145 is downregulated in lung carcinoma BMs, compared with primary tumors. However, there is no difference in miR-145 expression between tumors that can or cannot metastasize to the brain. The downregulation of miR-145 is caused by the methylation of its promoter and is followed by the upregulation of EGFR, octamer-binding transcription factor 4 (OCT4), mucin 1 (MUC1), c-Myc, and tumor protein D52 (TPD52). Therefore, lower levels of miR-145 lead to higher proliferation of cell lines derived from lung adenocarcinomas [125,126]. In cells of lung adenocarcinoma with brain tropism, miR-95-3p is downregulated together with inversely upregulated cyclin-D. MiR-95-3p can directly regulate cyclin D1 levels, with lower levels of cyclin D1 leading to lower invasiveness, colony formation, and a proliferation of lung cancer cell lines with brain tropism. Higher levels of miR-95-3p also suppress the formation of BMs of lung adenocarcinoma and prolong overall survival (OS) and brain metastasis free survival (BMFS) [127].

#### 5.1.2. Metastases Promoting MicroRNAs in Lung Cancer

Oncogenic miRNAs involved in the formation and development of lung carcinoma BMs are less described in the literature. MiR-378 is significantly overexpressed in NSCLC that further metastasizes to the brain. The overexpression of miR-378 contributes to survival, migration, and invasiveness, mainly due to the higher expression of VEGF, MMP2, and MMP9 and the downregulation of suppressor of fused homolog (SUFU). MiR-378 is also involved in the formation of vasculogenic mimicry [128]. In addition, miR-328 is upregulated in lung carcinoma that metastasizes to the brain, concurrently in primary tumor and secondary loci. The higher expression of miR-328 is responsible for the higher migratory capacity of primary tumor cells [129]. Patients with a higher expression of miR-143-3p typically have worse OS, and the expression of this miRNA is correlated with the occurrence of BMs and the overall progression of the disease. MiR-143-3p causes cell migration, invasiveness, and phenotype changes that are accompanied by the higher expression of mesenchymal markers in vitro. Furthermore, the downregulation of the direct target of miR-143-3p, vasohibin 1 (VASH1), causes decreased ubiquitylation of vascular endothelial growth factor A (VEGFA) and depolymerization of tubulin. The overexpression of miR-143-3p also has a tremendous impact on invasiveness and on the passage through the BBB, which supports the dissemination of lung cancer cells into the brain [130].

#### 5.1.3. MicroRNAs with Diagnostic and Prognostic Potential in Lung Cancer Metastases

Some miRNAs were identified as valuable diagnostic and prognostic biomarkers in lung carcinoma BMs, such as miR-let-7a, miR-330-3p, and miR-375. MiR-let-7a shows lower expression in the serum of patients with lung cancer with developed BMs, compared with the expression in patients without BMs. The higher level of miR-let-7a in serum is also a favorable factor for the efficiency of radiotherapy in patients with BMs. MiR-let-7a is also capable of reducing the proliferation of lung cancer cells in vitro [131]. MiR-330-3p significantly differentiates lung carcinoma patients with developed BMs from patients without them. At the same time, miR-330-3p can predict the formation of BMs. A higher expression of miR-330-3 causes higher proliferation, migration, invasiveness, and angiogenesis, as well as lower apoptosis in vitro. Higher levels of miR-330-3p lead to the promotion of tumorigenesis and BMs formation in vivo. The direct target of miR-330-3p is glutamate ionotropic receptor alpha-amino-3-hydroxy-5-methyl-4-isoxazole propionate type subunit 3 (GRIA3); a higher expression of GRIA3 causes a lower expression of TGF-β1, which is the main regulator of EMT. Therefore, miR-330-3p significantly influences the ability of lung cancer cells to develop metastasis; its inhibition leads to the inhibition of EMT through the miR-330-3p/GRIA/TGF-β1 pathway [132]. MiR-375 is downregulated in NSCLC that forms BMs, in comparison with tumors that do not form BMs. A significant downregulation of miR-375 is correlated with the advanced stage of the disease and the number of BMs, and is also correlated with poorer OS [133].

Another miRNA, miR-1207-5p, has a specific role in the development of BMs in lung cancer patients. The inhibition of this miRNA by the long ncRNA (lncRNA) lnc-MMP2-2 causes significantly lower expression of the endothelial marker vascular endothelial (VE)-cadherin and proteins of tight junctions, such as ZO-1, claudin-5, and occludin. In contrast, the mesenchymal marker N-cadherin is upregulated in BBB cells. All of these alterations in gene expression led to a higher permeability of the BBB, allowing easier penetration into the brain. The main mechanism is the upregulation of erythrocyte membrane protein band 4.1 Like 5 (EPB41L5), which is a direct target of miR-1207-5p. EPB41L5 further promotes endothelial-mesenchymal transition, destroying tight junctions, and inducing the permeability of the human brain microvascular endothelial cell (HBMEC) monolayer. Lnc-MMP-2 is exported through cancer cells exosomes into BBB cells and acts as a sponge for the protective miR-1207-5p. Therefore, cancer cells can indirectly influence cells that form the barrier and disrupt their ability to prevent cancer cells from entering the brain [134].

### 5.2. MicroRNAs in Breast Cancer Brain Metastases

Breast carcinomas are distinguished by the expression of receptors on the carcinoma cells’ surfaces. Hormone receptor-positive tumors express receptors for estrogen and progesterone. HER2+ tumors express HER2 receptors. If none of these receptors are expressed, the tumors are classified as TNBC, with the worst possible prognosis [135]. The most common targets for breast carcinoma metastases are the lungs, the liver, and the brain, followed by the bones and the skin [136]. Among patients with breast cancer, 15% to 25% develop metastases in the CNS [137]. The largest proportion of BMs is found in the brain parenchyma (78%), usually in the form of multiple metastases, although solitary metastases are also found [138]. Significant differences are observed in the tendencies of different subtypes of breast carcinoma to metastasize to the brain. The greatest inclination to metastasize in the brain is seen in HER2 + and TNBC tumors [139].

With a few exceptions, the differences in the expression of specific miRNAs between various subtypes of metastasizing breast tumors, with different organ tropisms, are not known. Nevertheless, the identification of miRNAs that differentiate between tumors that are likely to metastasize to the brain could have a huge diagnostic impact. However, there are a few obstacles in the way to identifying robust biomarkers for monitoring of the metastasizing processes. One complication is that miRNA profiles are rather dynamic and change throughout the progression of the disease. Some miRNAs that are dysregulated in the beginning of a metastatic cascade are not dysregulated in later stages. Luckily, some miRNAs seem to follow a steady trend of expression in the whole process of metastases development. For example, miR-802-5p and miR-194-5p, are downregulated in blood plasma in the early stages of the metastatic cascade, long before the development of brain macrometastases. Their common target is myocyte enhancer factor 2C (MEF2C), which is highly expressed in BMs; its expression level increases with the size of the macrometastases. MEF2C is a transcription factor with downstream targets, such as MMP10 and VEGF [140]. The study by Figueira et al., described not only the downregulation of miR-802-5p and miR-194-5p, but also the upregulation of miR-92a-1-5p, miR-205-5p, and miR-181a-1-3p; they also confirmed that miRNA levels in blood plasma correlate with their expression in BMs. While miR-205-5p is upregulated solely in metastasizing cells, miR-194-5p is downregulated not only in metastasizing cells but also in BBB cells, suggesting, perhaps, an important role in microenvironment modulation. Notably, the interactions between metastasizing cells and cells of the BBB are responsible for the upregulation of miR-181a-1-3p, suggesting a possible important role of this miRNA in the interactions between cancer cells and BBB. The mechanism and the reasons behind the downregulation of miR-802-5p are currently unknown [141].

Debeb et al., described the involvement of some of the previously mentioned miRNAs from the miR-200 family in the formation of breast BMs. Breast cancer cells metastasizing to the brain have a different, more epithelial-like phenotype and a higher expression of E-cadherin, which could be an important factor for migration toward the brain. Therefore, miRNAs promoting E-cadherin expression, such as those in the miR-200 family, could play an important role in metastasis tropism. MiR-200 highly influences E-cadherin expression. Therefore, this miRNA could potentially be highly impactful in the formation of BMs. Despite this, the knockdown of miR-200a has not shown a significant impact on the frequency of BMs formation, unlike the knockdown of miR-141, which, however, does not affect the formation of lung metastases. Higher levels of miR-141 in serum are associated with poorer OS and progression-free survival in patients with metastatic breast cancer [142].

#### 5.2.1. MicroRNAs with a Suppresive Function for Metastases Development in Breast Cancer

In the context of breast cancer, the downregulation of miR-7 could significantly contribute to the metastatic potential of breast cancer stem cells. A low expression of miR-7 is typical for mammospheres; it is assumed that lower levels of miR-7 are responsible for the self-renewal of cancer stem cells (CSC). MiR-7 targets KLF4, a crucial regulator of CSC stemness, regulating the expression of the TGF-β1 and Notch pathways. In vivo upregulation of miR-7-2 was shown to lead to a significant decrease of BM formation, with no effect on the formation of bone metastases [143]. Similarly, miR-146a is downregulated in breast carcinoma cell lines with higher tropism for the brain, compared with cell lines that do not metastasize into the brain. Lower levels of miR-146a lead to increased cell migration and invasiveness. In contrast, the upregulation of miR-146a is accompanied by an increased expression of β-catenin and a downregulation of heterogeneous nuclear ribonucleoprotein C (hnRNPC). The downregulation of hnRNPC is connected with the suppression of the Akt pathway, as well as with the lower expression of key MMPs, leading to the inhibition of BM development [144].

In primary tumors, miR-509 is highly expressed. However, as the metastatic cascade progresses, miR-509 must be silenced, as it regulates two essential genes for metastasizing— Ras homolog family member C (RhoC) and tumor necrosis factor α (TNFα). A higher expression of RhoC and TNFα causes a higher expression of MMP9 and a higher permeability of BBB. The downregulation of miR-509 is, therefore, important for the extravasation process and leads to increased invasiveness of metastasizing cells [145]. Another miRNA, miR-211, probably acts in the context of extravasation and is upregulated in cells with brain tropism, but its expression is significantly higher in the brain, compared with the primary loci. The upregulation of miR-211 also leads to poor survival and a higher number of metastases in vivo. MiR-211 increases the ability of cancer cells to adhere to the BBB and simultaneously increases their transmigration capacity through the BBB. In addition, miR-211 probably supports the stemness of cells, as it is upregulated in CSCs and spheres in vitro. The target genes are SOX11 and neurogenin 2 (NGN2), whose expression prevents higher adhesion to the BBB, development of metastases, and survival [146].

#### 5.2.2. MicroRNAs Involved in the Extravasation and Colonization of the Brain in Breast Cancer Metastases

In the process of disrupting the BBB and extravasation, exosomes play a crucial role. MiR-181c, which is secreted in exosomes derived from breast cancer cells, causes the destruction of the BBB by delocalizing actin filaments, due to the downregulation of phosphoinositide-dependent kinase 1 (PDPK1). Malfunctions in the localization of actin filaments lead to abnormally tight junctions and the destruction of cell-to-cell contacts. Extracellular vesicles produced by circulating cancer cells could be one of the main causes of the formation of BMs, as they are able to completely disrupt BBB and allow extravasation in the brain. While miR-181c is not upregulated in the primary tumor, higher levels of miR-181c were detected in blood plasma of BM patients [147]. Another exosome-contained miRNA involved in the metastatic cascade is miR-503, which promotes the conversion of M1-M2 in microglia and modifies the brain microenvironment, allowing cells to attach and grow more easily. This action is boosted by the increased expression of programmed death-ligand 1 (PD-L1) in microglia, suppressing the immune surveillance and allowing tumor cells to spread into the brain [148].

An interesting mechanism of action was observed when PTEN was lost in breast cancer BMs. While the loss of PTEN is typical for some primary brain tumors, it is found in breast cells only when it is in the cells that metastasize into the brain. If breast cancer cells subsequently intravasate from the secondary loci in the brain, the PTEN expression is restored. Therefore, the loss of PTEN is not caused by cancer cells themselves, but by the brain microenvironment, which, therefore, plays a crucial role in its downregulation. Specifically, miR-19a, which is contained in astrocyte-secreted exosomes, causes the downregulation of PTEN, which subsequently leads to the activation of the NF-κB and Akt pathways and to an increased expression of C-C motif chemokine ligand 2 (CCL2), followed by the recruitment of ionized calcium-binding adapter molecule 1 (IBA-1)-expressing myeloid cells. MiR-19a secretion is, thus, responsible for the promotion of BM growth, proliferation, and the reduction of apoptosis [15]. Additionally, miR-122 is secreted in the form of extracellular vesicles and causes a decrease in glucose uptake and a lower expression of glucose transporter 1 (GLUT1) and pyruvate kinase isozyme M2 (PKM2) in the target cells, as miR-122 targets pyruvate kinase and citrate kinase. Astrocytes that receive those vesicles suffer from subdued glucose uptake, while metastasizing cells utilize a higher amount of glucose, causing them to proliferate more intensively. Therefore, miR-122 can prime the premetastatic niche and support the colonization and formation of new metastases [149].

### 5.3. MicroRNAs in Melanoma Brain Metastases

Cancers that do not metastasize to the brain as often as lung or breast carcinomas receive less scientific interest, which is also the case with the research on miRNAs’ influence on the formation of their BMs. However, several studies have attempted to elucidate the roles of specific miRNAs in the pathology of BMs that are derived from melanoma, CRC, and RCC. Of these three, melanoma is the most frequently associated with the formation of BM. The incidence of melanoma is constantly rising, and among all skin cancers worldwide, melanoma is responsible for most deaths. Melanoma arises from melanocytes, a minor population of skin cells with very low proliferation that is responsible for melanin production. Soon after the formation of melanoma, small tumors may already have a high metastatic potential [150].

Generally, in melanoma metastases, the miRNA cluster miR-224-5p/miR-452 was described as responsible for EMT induction and cytoskeletal conversion, increased migration capacity, and invasiveness. MiRNAs from the cluster miR-224-5p/miR-452 target tumor suppressor of metastases thioredoxin-interacting protein (TXNIP). The downregulation of TXNIP is important for the E2F transcription factor 1 (E2F1)-mediated induction of EMT [151]. Another miRNA, miR-542-3p, which targets serine/threonine protein kinase Pim-1, is downregulated in melanoma metastases. The upregulation of this miRNA results in the inhibition of cell migration, invasion, and EMT [152].

The study by Mikkelsen et al., described global miRNA profiling with the aim of finding miRNAs that could significantly differentiate between metastasizing and non-metastasizing melanomas. They identified six downregulated miRNAs (miR-34a-3p, miR-548f-4, miR-1270, miR-1290, miR-4278, and miR-4528) and nine upregulated miRNAs (miR-518a-5p, miR-527, miR-575, miR-622, miR-4501, miR-4654, miR-4698, miR-6759-5p, and miR-8078) in metastasizing melanomas, compared their non-metastasizing counterparts. Six miRNAs were described as significantly dysregulated between distant metastases and primary tumor tissue (miR-184, miR-302d-5p, miR-658, miR-1246, miR-4427, and miR-3084) [153].

Bustos et al., dealt with the identification of circulating cell-free miRNAs that differentiated between patients with metastasizing melanoma and healthy controls, revealing 29 circulating miRNAs that are deregulated in metastasizing melanoma. They also compared the miRNA expression profiles in the plasma of BMs of lung and breast cancer, glioblastoma patients’ plasma, and melanoma BMs patients’ plasma. Six miRNAs were specific for melanoma BMs (miR-671-5p, miR-4664-3p, miR-4665-3p, miR-5694, miR-6741-3p, and miR-6796-3p) [154]. A study by Hanniford et al., dealt with the prediction of the formation of BMs from melanoma. After comparing melanomas with developed BMs and those without BMs, they identified four significantly dysregulated miRNAs (miR-15b-5p, miR-16-5p, miR-150-5p, and miR-374b-3p). Subsequently, miR-150-5p was described as involved in the suppression of cell proliferation, migration, and invasiveness through sine oculis homeobox homolog 1 (SIX1) inhibition. The downregulation of SIX1 causes limitations of glycolysis, due to the decrease in glucose uptake, lactate production, or adenosine triphosphate (ATP) generation [155,156].

### 5.4. MicroRNAs in Colorectal Carcinoma Brain Metastases

CRC is the most common cancer affecting the digestive tract. Despite more advanced therapy approaches and longer patient survival, BMs are becoming a more common problem in the treatment of CRC. Although BMs in CRC are relatively rare compared with the diagnoses mentioned above, it is estimated that up to 1% to 4% of patients with CRC develop BMs. Moreover, the incidence of BMs in CRC is increasing over time [157].

Li et al., studied the miRNA expression in primary tumors and CRCs that metastasize into the brain, finding 19 dysregulated miRNAs and 17 upregulated in BMs. MiR-125b was further analyzed by RT-qPCR, and its upregulation in CRC BMs was verified. However, several issues arose with this study, mainly due to a relatively small cohort of patients, so the results were not sufficient to consider in the context of CRC [158]. However, some of the miRNAs that were analyzed, such as miR-145 and miR-31, were also described in other brain malignancies, such as glioblastoma, suggesting a possible role in the pathology of brain tumors [159,160]. Additionally, miR-590-5p is among miRNAs that are potentially responsible for metastatic reversal in CRC. It suppresses reversion inducing cysteine rich protein with Kazal motifs (RECK), and inversely upregulates levels of focal adhesion kinase (FAK), Akt, and Rac family small GTPase 1 (RAC1), leading to the formation of tumor protrusions and increasing cell mobility. This miRNA is sensitive to hypoxia and could be responsible for the acquisition of abilities that allow CRC cells to metastasize [161].

### 5.5. MicroRNAs in Renal Cell Carcinoma Brain Metastases

RCC is a malignant carcinoma of the kidney and represents around 3% of all malignant tumors [162]. The most common subtype of RCC is clear cell RCC (ccRCC), which accounts for approximately 70% of all RCCs. Other subtypes are papillary RCC, chromophobe RCC, nephron and collecting system RCC, and non-classified RCC [163]. Although RCC is the fourth most common cancer that metastasizes to the brain, currently only one general article is available on the involvement of miRNAs in the process.

In the context of RCC metastasis, miR-10a is responsible for the downregulation of BDNF, which otherwise supports the expression of MMPs and, therefore, plays a crucial role in RCC metastases [164]. Furthermore, Heinzelmann et al., identified miR-10b as downregulated in the BMs of RCC, compared with primary tumor tissues. Among other analyzed miRNAs, miR-30c was the most downregulated in RCC BMs, compared with normal kidney tissue, nonmetastatic primary ccRCC, and other metastatic primary ccRCC [165].

Several other miRNAs could be related to the metastatic behavior of RCC. MiR-206 is significantly downregulated in primary tumor tissue compared with adjacent non-tumor tissue; moreover, lower levels of miR-206 relate to the onset of BMs. An increase in miR-206 levels leads to decreased invasiveness and migration of cancer cells, targeting VEGFA, potent molecules that are connected to metastatic cascade [166]. MiR-384 is also downregulated, as it targets astrocyte elevated gene 1 (AEG1), the downstream target of Ha-ras. The upregulation of miR-384 leads to the suppression of invasiveness and migration of RCC cells, as it influences Wnt signaling [167]. In addition, the downregulation of miR-588 leads to higher expression of eukaryotic translation initiation factor 5A2 (EIF5A2), causing higher migration, invasiveness, and metastatic potential [168].

The list of miRNAs specifically deregulated in brain metastases is shown in Table 1.

## 6. Conclusions

BMs are a relatively common and destructive event in the later stages of cancer that severely affect patients’ the quality of life. During the last few years, the incidence of BMs has increased, mainly due to more efficient therapies and the prolonged survival of cancer patients, which lead to longer times for metastasizing cells to penetrate the blood-brain barrier and colonize the brain. To predict the risk of the formation of BMs in patients with tumors that are known to metastasize frequently toward the brain, new predictive and diagnostic biomarkers are urgently needed. The current diagnostic methods are not able to uncover metastasizing cells or circulating cancer cells; however, there is great potential for identifying molecules that could be responsible for the origin of metastasis development. Their dysregulated expression could be a valuable predictor of the progression of the disease. Among these molecules, small RNA regulatory molecules, such as microRNAs, seem to be very promising, as they are potent and stable regulators of crucial biological processes. MiRNAs are often dysregulated in various cancers, with some being described in the context of the metastatic cascade in different types of tumors. Therefore, the identification of miRNAs with the ability to differentiate tumors that are prone to metastasizing to the brain will be greatly beneficial for patients with BMs. In addition, miRNAs that can distinguish BMs from the tumors of primary origin could be a cornerstone for more personalized treatment of BMs patients, even in cases of unknown origin. All in all, the current evidence suggests that miRNAs are potent players in the metastatic cascade and may serve as promising biomarkers or therapeutic targets for patients with brain metastases.

## Figures and Tables

**Table 1:** MicroRNAs specifically dysregulated in brain metastases originating from specific primary tumors (LC = lung carcinoma, NSCLC = non-small cell lung carcinoma, K-Ras = protein encoded by Kirsten rat sarcoma virus (*KRAS*) oncogene, BBB = blood-brain barrier, EMT = epithelial-mesenchymal transition, BC = breast carcinoma, TNBC = triple negative breast carcinoma, CSC = cancer stem cells, BM = brain metastasis, HS = homo sapiens, CRC = colorectal carcinoma, RCC = renal cell carcinoma).

microRNA	Primary Tumor	Dysregulation	Role in Metastases	Origin	References
miR-21	LC, NSCLC	Up	Promotion of cell migration, invasion, proliferation, and angiogenesis	Cancer cells	[118,119,120]
miR-768-3p	LC	Dow	Brain colonization via K-Ras expression enhancement	Cancer cells	[121]
miR-193b	LC	Down	Inhibition of cell migration and invasion	Cancer cells	[122]
miR-215-3p	LC	Down	Not specified	Cancer cells	[123]
miR-217	LC, NSCLC	Down	Inhibition of cell viability and migration	Cancer cells	[124]
miR-145	LC	Down	Inhibition of cell migration	Cancer cells	[125,126]
miR-95-3p	LC	Down	Inhibition of cell proliferation and invasiveness via targeting Cyclin D1	Cancer Cells	[127]
miR-378	LC, NSCLC	Up	Promotion of cell migration, invasiveness and vasculogeny mimicry	Cancer cells	[128]
miR-328	LC, NSCLC	Up	Promotion of primary tumor cells migration	Cancer cells	[129]
miR-143-3p	LC	Up	Promotion of cell migration, invasiveness, and BBB passage	Cancer cells	[130]
let-7a	LC	Down	Inhibition of cell proliferation	Cancer cells	[131]
miR-330-3p	LC, NSCLC	Up	EMT promotion	Cancer cells	[132]
miR-375	LC, NSCLC	Down	Not examined	Cancer cells	[133]
miR-1207-5p	LC	Down	Promoting BBB permeability	Brain microvascular endothelial cells	[134]
miR-802-5p	BC, TNBC	Down	Not known	Not known	[140,141]
miR-194-5p	BC, TNBC	Down	Supposedly promotion of passage through BBB	Cancer cells, BBB cells	[140,141]
miR-92a-1-5p	BC, TNBC	Up	Not examined	Cancer cells, BBB cells	[141]
miR-205-5p	BC, TNBC	Up	Not examined	Cancer cells	[141]
miR-181a-1-3p	BC, TNBC	Up	Not examined	Cancer cells, BBB cells	[141]
miR-141	BC	Up	Not examined	Cancer cells	[142]
miR-7	BC	Down	Inhibition of CSC self-renrewal	CSC	[143]
miR-146a	BC	Down	Inhibition of cell migration and invasion	Cancer cells	[144]
miR-509	BC	Down	Inhibition of extravasation, BBB disruption	Upregulated in metastasizing cells	[145]
miR-211	BC, TNBC	Up	Promotion of passage through BBB	Upregulated in metastasizing cells	[146]
miR-181c	BC	Up	BBB disruption, Extravasation	Cancer cells (secreted via exosoms)	[147]
miR-503	BC	Up	Microenvironment modulation	Cancer cells (secreted via exosoms)	[148]
miR-19a	BC	Up	Promotion of BM growth, proliferation and inhibition of apoptosis	Astrocytes	[15]
miR-122	BC	Up	Microenvironment modulation	Cancer cells (secreted via exosoms)	[149]
miR-224-5p/miR-452 cluster	Melanoma	Up	EMT promotion	Cancer cells	[151]
miR-542-3p	Melanoma	Down	EMT Inhibition	Cancer cells	[152]
miR-671-5p, miR-4664-3p, miR-4665-3p, miR-5694, miR-6741-3p, and miR-6796-3p	Melanoma	Up	Not Examined	Not stated	[154]
miR-150-5p	Melanoma	Down	Inhibition of cell proliferation, migration and invasivness	Cancer cells	[155,156]
miR-199a, miR-133a, miR-145, miR-143, miR-10b, miR-1, miR-199a-5p, miR-145-3p, miR-125b, miR-133b, miR-22, miR-126-5p, miR-146a, miR-28-5p, miR-576-5p, miR-199b-5p, HS_287	CRC	Up	Not examined	Cancer cells	[158]
miR-31, HS_170	CRC	Down	Not examined	Cancer cells	[158]
miR-590-5p	CRC	Up	Cell mobility promotion	Cancer cells	[161]
miR-10b, miR-30c	RCC	Down	Not examined	Cancer cells	[165]
miR-206	RCC	Down	Inhibition of cell migration and invasiveness	Cancer cells	[166]

## References

[1] Nayak L., Lee E.Q., Wen P.Y. (2012). Epidemiology of Brain Metastases. Curr. Oncol. Rep..

[2] Smedby K.E., Brandt L., Bäcklund M.L., Blomqvist P. (2009). Brain Metastases Admissions in Sweden between 1987 and 2006. Br. J. Cancer.

[3] Niemiec M., Głogowski M., Tyc-Szczepaniak D., Wierzchowski M., Kępka L. (2011). Characteristics of Long-Term Survivors of Brain Metastases from Lung Cancer. Rep. Pract. Oncol. Radiother..

[4] Watabe K. (2016). Non-Coding RNAs in Cancer Brain Metastasis. Front. Biosci..

[5] Sperduto P.W., Kased N., Roberge D., Xu Z., Shanley R., Luo X., Sneed P.K., Chao S.T., Weil R.J., Suh J. (2012). Summary Report on the Graded Prognostic Assessment: An Accurate and Facile Diagnosis-Specific Tool to Estimate Survival for Patients with Brain Metastases. J. Clin. Oncol..

[6] Barnholtz-Sloan J.S., Sloan A.E., Davis F.G., Vigneau F.D., Lai P., Sawaya R.E. (2004). Incidence Proportions of Brain Metastases in Patients Diagnosed (1973 to 2001) in the Metropolitan Detroit Cancer Surveillance System. J. Clin. Oncol..

[7] Schouten L.J., Rutten J., Huveneers H.A.M., Twijnstra A. (2002). Incidence of Brain Metastases in a Cohort of Patients with Carcinoma of the Breast, Colon, Kidney, and Lung and Melanoma. Cancer.

[8] Ostrom Q.T., Wright C.H., Barnholtz-Sloan J.S. (2018). Brain Metastases: Epidemiology. Handbook of Clinical Neurology.

[9] Cagney D.N., Martin A.M., Catalano P.J., Redig A.J., Lin N.U., Lee E.Q., Wen P.Y., Dunn I.F., Bi W.L., Weiss S.E. (2017). Incidence and Prognosis of Patients with Brain Metastases at Diagnosis of Systemic Malignancy: A Population-Based Study. Neuro-Oncol..

[10] Martin A.M., Cagney D.N., Catalano P.J., Warren L.E., Bellon J.R., Punglia R.S., Claus E.B., Lee E.Q., Wen P.Y., Haas-Kogan D.A. (2017). Brain Metastases in Newly Diagnosed Breast Cancer: A Population-Based Study. JAMA Oncol..

[11] Ramakrishna N., Temin S., Chandarlapaty S., Crews J.R., Davidson N.E., Esteva F.J., Giordano S.H., Gonzalez-Angulo A.M., Kirshner J.J., Krop I. (2014). Recommendations on Disease Management for Patients with Advanced Human Epidermal Growth Factor Receptor 2–Positive Breast Cancer and Brain Metastases: American Society of Clinical Oncology Clinical Practice Guideline. J. Clin. Oncol..

[12] Langley R.R., Fidler I.J. (2011). The Seed and Soil Hypothesis Revisited-The Role of Tumor-Stroma Interactions in Metastasis to Different Organs. Int. J. Cancer.

[13] Kuzet S.-E., Gaggioli C. (2016). Fibroblast Activation in Cancer: When Seed Fertilizes Soil. Cell Tissue Res..

[14] Kaplan R.N., Riba R.D., Zacharoulis S., Bramley A.H., Vincent L., Costa C., MacDonald D.D., Jin D.K., Shido K., Kerns S.A. (2005). VEGFR1-Positive Haematopoietic Bone Marrow Progenitors Initiate the Pre-Metastatic Niche. Nature.

[15] Zhang L., Zhang S., Yao J., Lowery F.J., Zhang Q., Huang W.-C., Li P., Li M., Wang X., Zhang C. (2015). Microenvironment-Induced PTEN Loss by Exosomal MicroRNA Primes Brain Metastasis Outgrowth. Nature.

[16] Draffin J.E., McFarlane S., Hill A., Johnston P.G., Waugh D.J.J. (2004). CD44 Potentiates the Adherence of Metastatic Prostate and Breast Cancer Cells to Bone Marrow Endothelial Cells. Cancer Res..

[17] Brabletz T. (2012). To Differentiate or Not—Routes towards Metastasis. Nat. Rev. Cancer.

[18] Rettig M., Trinidad K., Pezeshkpour G., Frost P., Sharma S., Moatamed F., Tamanoi F., Mortazavi F. (2012). PAK1 Kinase Promotes Cell Motility and Invasiveness through CRK-II Serine Phosphorylation in Non-Small Cell Lung Cancer Cells. PLoS ONE.

[19] Craene B.D., Berx G. (2013). Regulatory Networks Defining EMT during Cancer Initiation and Progression. Nat. Rev. Cancer.

[20] Mani S.A., Guo W., Liao M.-J., Eaton E.N., Ayyanan A., Zhou A.Y., Brooks M., Reinhard F., Zhang C.C., Shipitsin M. (2008). The Epithelial-Mesenchymal Transition Generates Cells with Properties of Stem Cells. Cell.

[21] Simpson C.D., Anyiwe K., Schimmer A.D. (2008). Anoikis Resistance and Tumor Metastasis. Cancer Lett..

[22] Luzzi K.J., MacDonald I.C., Schmidt E.E., Kerkvliet N., Morris V.L., Chambers A.F., Groom A.C. (1998). Multistep Nature of Metastatic Inefficiency. Am. J. Pathol..

[23] Thiery J.P., Acloque H., Huang R.Y.J., Nieto M.A. (2009). Epithelial-Mesenchymal Transitions in Development and Disease. Cell.

[24] Wang Y., Bu F., Royer C., Serres S., Larkin J.R., Soto M.S., Sibson N.R., Salter V., Fritzsche F., Turnquist C. (2014). ASPP2 Controls Epithelial Plasticity and Inhibits Metastasis through β-Catenin-Dependent Regulation of ZEB1. Nat. Cell Biol..

[25] Díaz-López A., Díaz-Martín J., Moreno-Bueno G., Cuevas E.P., Santos V., Olmeda D., Portillo F., Palacios J., Cano A. (2015). Zeb1 and Snail1 Engage MiR-200f Transcriptional and Epigenetic Regulation during EMT: EMT Players Controlling Epithelial Plasticity. Int. J. Cancer.

[26] Sun T., Zhao N., Zhao X., Gu Q., Zhang S., Che N., Wang X., Du J., Liu Y., Sun B. (2010). Expression and Functional Significance of Twist1 in Hepatocellular Carcinoma: Its Role in Vasculogenic Mimicry. Hepatology.

[27] Ocaña O.H., Córcoles R., Fabra Á., Moreno-Bueno G., Acloque H., Vega S., Barrallo-Gimeno A., Cano A., Nieto M.A. (2012). Metastatic Colonization Requires the Repression of the Epithelial-Mesenchymal Transition Inducer Prrx1. Cancer Cell.

[28] Wang J., He H., Jiang Q., Wang Y., Jia S. (2020). CBX6 Promotes HCC Metastasis Via Transcription Factors Snail/Zeb1-Mediated EMT Mechanism. Onco Targets Ther..

[29] Morrison C.D., Parvani J.G., Schiemann W.P. (2013). The Relevance of the TGF-β Paradox to EMT-MET Programs. Cancer Lett..

[30] Tan E.-J., Olsson A.-K., Moustakas A. (2015). Reprogramming during Epithelial to Mesenchymal Transition under the Control of TGFβ. Cell Adhes. Migr..

[31] VanderVorst K., Dreyer C.A., Konopelski S.E., Lee H., Ho H.-Y.H., Carraway K.L. (2019). Wnt/PCP Signaling Contribution to Carcinoma Collective Cell Migration and Metastasis. Cancer Res..

[32] Li L., Tang P., Li S., Qin X., Yang H., Wu C., Liu Y. (2017). Notch Signaling Pathway Networks in Cancer Metastasis: A New Target for Cancer Therapy. Med. Oncol..

[33] Joseph J.P., Harishankar M.K., Pillai A.A., Devi A. (2018). Hypoxia Induced EMT: A Review on the Mechanism of Tumor Progression and Metastasis in OSCC. Oral Oncol..

[34] Brizel D.M., Schroeder T., Scher R.L., Walenta S., Clough R.W., Dewhirst M.W., Mueller-Klieser W. (2001). Elevated Tumor Lactate Concentrations Predict for an Increased Risk of Metastases in Head-and-Neck Cancer. Int. J. Radiat. Oncol..

[35] Goetze K. (2011). Lactate Enhances Motility of Tumor Cells and Inhibits Monocyte Migration and Cytokine Release. Int. J. Oncol..

[36] Knudsen E.S., Ertel A., Davicioni E., Kline J., Schwartz G.F., Witkiewicz A.K. (2012). Progression of Ductal Carcinoma in Situ to Invasive Breast Cancer Is Associated with Gene Expression Programs of EMT and Myoepithelia. Breast Cancer Res. Treat..

[37] Thiery J.P. (2002). Epithelial–Mesenchymal Transitions in Tumour Progression. Nat. Rev. Cancer.

[38] Wang Y., Zhou B.P. (2011). Epithelial-Mesenchymal Transition in Breast Cancer Progression and Metastasis. Chin. J. Cancer.

[39] Roh M.R., Zheng Z., Kim H.S., Kwon J.E., Jeung H.-C., Rha S.Y., Chung K.Y. (2012). Differential Expression Patterns of MMPs and Their Role in the Invasion of Epithelial Premalignant Tumors and Invasive Cutaneous Squamous Cell Carcinoma. Exp. Mol. Pathol..

[40] Rahman M., Mohammed S. (2015). Breast Cancer Metastasis and the Lymphatic System. Oncol. Lett..

[41] Wong A.D., Searson P.C. (2014). Live-Cell Imaging of Invasion and Intravasation in an Artificial Microvessel Platform. Cancer Res..

[42] Bolós V., Mira E., Martínez-Poveda B., Luxán G., Cañamero M., Martínez-A C., Mañes S., de la Pompa J.L. (2013). Notch Activation Stimulates Migration of Breast Cancer Cells and Promotes Tumor Growth. Breast Cancer Res..

[43] Sonoshita M., Aoki M., Fuwa H., Aoki K., Hosogi H., Sakai Y., Hashida H., Takabayashi A., Sasaki M., Robine S. (2011). Suppression of Colon Cancer Metastasis by Aes through Inhibition of Notch Signaling. Cancer Cell.

[44] Khuon S., Liang L., Dettman R.W., Sporn P.H.S., Wysolmerski R.B., Chew T.-L. (2010). Myosin Light Chain Kinase Mediates Transcellular Intravasation of Breast Cancer Cells through the Underlying Endothelial Cells: A Three-Dimensional FRET Study. J. Cell Sci..

[45] Arvanitis C., Khuon S., Spann R., Ridge K.M., Chew T.-L. (2014). Structure and Biomechanics of the Endothelial Transcellular Circumferential Invasion Array in Tumor Invasion. PLoS ONE.

[46] Labelle M., Hynes R.O. (2012). The Initial Hours of Metastasis: The Importance of Cooperative Host–Tumor Cell Interactions during Hematogenous Dissemination. Cancer Discov..

[47] Mamessier E., Sylvain A., Thibult M.-L., Houvenaeghel G., Jacquemier J., Castellano R., Gonçalves A., André P., Romagné F., Thibault G. (2011). Human Breast Cancer Cells Enhance Self Tolerance by Promoting Evasion from NK Cell Antitumor Immunity. J. Clin. Investig..

[48] Moose D.L., Krog B.L., Kim T.-H., Zhao L., Williams-Perez S., Burke G., Rhodes L., Vanneste M., Breheny P., Milhem M. (2020). Cancer Cells Resist Mechanical Destruction in Circulation via RhoA/Actomyosin-Dependent Mechano-Adaptation. Cell Rep..

[49] Kopp H.-G., Placke T., Salih H.R. (2009). Platelet-Derived Transforming Growth Factor-β Down-Regulates NKG2D Thereby Inhibiting Natural Killer Cell Antitumor Reactivity. Cancer Res..

[50] Kienast Y., von Baumgarten L., Fuhrmann M., Klinkert W.E.F., Goldbrunner R., Herms J., Winkler F. (2010). Real-Time Imaging Reveals the Single Steps of Brain Metastasis Formation. Nat. Med..

[51] Valiente M., Obenauf A.C., Jin X., Chen Q., Zhang X.H.-F., Lee D.J., Chaft J.E., Kris M.G., Huse J.T., Brogi E. (2014). Serpins Promote Cancer Cell Survival and Vascular Co-Option in Brain Metastasis. Cell.

[52] Liu Y., Kosaka A., Ikeura M., Kohanbash G., Fellows-Mayle W., Snyder L.A., Okada H. (2013). Premetastatic Soil and Prevention of Breast Cancer Brain Metastasis. Neuro-Oncol..

[53] Hanibuchi M., Kim S.-J., Fidler I.J., Nishioka Y. (2014). The Molecular Biology of Lung Cancer Brain Metastasis: An Overview of Current Comprehensions and Future Perspectives. J. Med. Investig..

[54] Soto M.S., Serres S., Anthony D.C., Sibson N.R. (2014). Functional Role of Endothelial Adhesion Molecules in the Early Stages of Brain Metastasis. Neuro-Oncol..

[55] Wu K., Fukuda K., Xing F., Zhang Y., Sharma S., Liu Y., Chan M.D., Zhou X., Qasem S.A., Pochampally R. (2015). Roles of the Cyclooxygenase 2 Matrix Metalloproteinase 1 Pathway in Brain Metastasis of Breast Cancer. J. Biol. Chem..

[56] Gunasinghe N.P.A.D., Wells A., Thompson E.W., Hugo H.J. (2012). Mesenchymal–Epithelial Transition (MET) as a Mechanism for Metastatic Colonisation in Breast Cancer. Cancer Metastasis Rev..

[57] Yoshida T., Ozawa Y., Kimura T., Sato Y., Kuznetsov G., Xu S., Uesugi M., Agoulnik S., Taylor N., Funahashi Y. (2014). Eribulin Mesilate Suppresses Experimental Metastasis of Breast Cancer Cells by Reversing Phenotype from Epithelial–Mesenchymal Transition (EMT) to Mesenchymal–Epithelial Transition (MET) States. Br. J. Cancer.

[58] Chao Y., Wu Q., Acquafondata M., Dhir R., Wells A. (2012). Partial Mesenchymal to Epithelial Reverting Transition in Breast and Prostate Cancer Metastases. Cancer Microenviron..

[59] Wanner I.B., Anderson M.A., Song B., Levine J., Fernandez A., Gray-Thompson Z., Ao Y., Sofroniew M.V. (2013). Glial Scar Borders Are Formed by Newly Proliferated, Elongated Astrocytes That Interact to Corral Inflammatory and Fibrotic Cells via STAT3-Dependent Mechanisms after Spinal Cord Injury. J. Neurosci..

[60] Lorger M., Felding-Habermann B. (2010). Capturing Changes in the Brain Microenvironment during Initial Steps of Breast Cancer Brain Metastasis. Am. J. Pathol..

[61] Fitzgerald D.P., Palmieri D., Hua E., Hargrave E., Herring J.M., Qian Y., Vega-Valle E., Weil R.J., Stark A.M., Vortmeyer A.O. (2008). Reactive Glia Are Recruited by Highly Proliferative Brain Metastases of Breast Cancer and Promote Tumor Cell Colonization. Clin. Exp. Metastasis.

[62] Xing F., Kobayashi A., Okuda H., Watabe M., Pai S.K., Pandey P.R., Hirota S., Wilber A., Mo Y., Moore B.E. (2013). Reactive Astrocytes Promote the Metastatic Growth of Breast Cancer Stem-like Cells by Activating Notch Signalling in Brain. EMBO Mol. Med..

[63] Ye X., Xu S., Xin Y., Yu S., Ping Y., Chen L., Xiao H., Wang B., Yi L., Wang Q. (2012). Tumor-Associated Microglia/Macrophages Enhance the Invasion of Glioma Stem-like Cells via TGF-Β1 Signaling Pathway. J. Immunol..

[64] Demeule M., Bertrand Y., Michaud-Levesque J., Jodoin J., Rolland Y., Gabathuler R., Béliveau R. (2003). Regulation of Plasminogen Activation: A Role for Melanotransferrin (P97) in Cell Migration. Blood.

[65] Dunn L.L., Sekyere E.O., Suryo Rahmanto Y., Richardson D.R. (2006). The Function of Melanotransferrin: A Role in Melanoma Cell Proliferation and Tumorigenesis. Carcinogenesis.

[66] Kim S.W., Choi H.J., Lee H.-J., He J., Wu Q., Langley R.R., Fidler I.J., Kim S.-J. (2014). Role of the Endothelin Axis in Astrocyte- and Endothelial Cell-Mediated Chemoprotection of Cancer Cells. Neuro-Oncol..

[67] Chen Q., Boire A., Jin X., Valiente M., Er E.E., Lopez-Soto A., Jacob L.S., Patwa R., Shah H., Xu K. (2016). Carcinoma–Astrocyte Gap Junctions Promote Brain Metastasis by CGAMP Transfer. Nature.

[68] Neman J., Termini J., Wilczynski S., Vaidehi N., Choy C., Kowolik C.M., Li H., Hambrecht A.C., Roberts E., Jandial R. (2014). Human Breast Cancer Metastases to the Brain Display GABAergic Properties in the Neural Niche. Proc. Natl. Acad. Sci. USA.

[69] Grupenmacher A.T., Halpern A.L., Bonaldo M.d.F., Huang C.-C., Hamm C.A., de Andrade A., Tomita T., Sredni S.T. (2013). Study of the Gene Expression and MicroRNA Expression Profiles of Malignant Rhabdoid Tumors Originated in the Brain (AT/RT) and in the Kidney (RTK). Childs Nerv. Syst..

[70] Huarte M., Rinn J.L. (2010). Large Non-Coding RNAs: Missing Links in Cancer?. Hum. Mol. Genet..

[71] International Human Genome Sequencing Consortium (2001). Initial Sequencing and Analysis of the Human Genome. Nature.

[72] Lu J., Getz G., Miska E.A., Alvarez-Saavedra E., Lamb J., Peck D., Sweet-Cordero A., Ebert B.L., Mak R.H., Ferrando A.A. (2005). MicroRNA Expression Profiles Classify Human Cancers. Nature.

[73] Volinia S., Calin G.A., Liu C.-G., Ambs S., Cimmino A., Petrocca F., Visone R., Iorio M., Roldo C., Ferracin M. (2006). A MicroRNA Expression Signature of Human Solid Tumors Defines Cancer Gene Targets. Proc. Natl. Acad. Sci. USA.

[74] MacRae I.J., Zhou K., Li F., Repic A., Brooks A.N., Cande W.Z., Adams P.D., Doudna J.A. (2006). Structural Basis for Double-Stranded RNA Processing by Dicer. Science.

[75] Kobayashi H., Tomari Y. (2016). RISC Assembly: Coordination between Small RNAs and Argonaute Proteins. Biochim. Biophys. Acta BBA Gene Regul. Mech..

[76] Chendrimada T.P., Gregory R.I., Kumaraswamy E., Norman J., Cooch N., Nishikura K., Shiekhattar R. (2005). TRBP Recruits the Dicer Complex to Ago2 for MicroRNA Processing and Gene Silencing. Nature.

[77] Meister G., Tuschl T. (2004). Mechanisms of Gene Silencing by Double-Stranded RNA. Nature.

[78] Eichhorn S.W., Guo H., McGeary S.E., Rodriguez-Mias R.A., Shin C., Baek D., Hsu S., Ghoshal K., Villén J., Bartel D.P. (2014). MRNA Destabilization Is the Dominant Effect of Mammalian MicroRNAs by the Time Substantial Repression Ensues. Mol. Cell.

[79] Lewis B.P., Shih I., Jones-Rhoades M.W., Bartel D.P., Burge C.B. (2003). Prediction of Mammalian MicroRNA Targets. Cell.

[80] Tay Y., Zhang J., Thomson A.M., Lim B., Rigoutsos I. (2008). MicroRNAs to Nanog, Oct4 and Sox2 Coding Regions Modulate Embryonic Stem Cell Differentiation. Nature.

[81] Miska E.A., Alvarez-Saavedra E., Abbott A.L., Lau N.C., Hellman A.B., McGonagle S.M., Bartel D.P., Ambros V.R., Horvitz H.R. (2007). Most Caenorhabditis Elegans MicroRNAs Are Individually Not Essential for Development or Viability. PLoS Genet..

[82] Bernstein E., Kim S.Y., Carmell M.A., Murchison E.P., Alcorn H., Li M.Z., Mills A.A., Elledge S.J., Anderson K.V., Hannon G.J. (2003). Dicer Is Essential for Mouse Development. Nat. Genet..

[83] Vidigal J.A., Ventura A. (2015). The Biological Functions of MiRNAs: Lessons from in Vivo Studies. Trends Cell Biol..

[84] Wang D., Zhang Z., O’Loughlin E., Wang L., Fan X., Lai E.C., Yi R. (2013). MicroRNA-205 Controls Neonatal Expansion of Skin Stem Cells by Modulating the PI(3)K Pathway. Nat. Cell Biol..

[85] Ventura A., Young A.G., Winslow M.M., Lintault L., Meissner A., Erkeland S.J., Newman J., Bronson R.T., Crowley D., Stone J.R. (2008). Targeted Deletion Reveals Essential and Overlapping Functions of the MiR-17∼92 Family of MiRNA Clusters. Cell.

[86] Calin G.A., Sevignani C., Dumitru C.D., Hyslop T., Noch E., Yendamuri S., Shimizu M., Rattan S., Bullrich F., Negrini M. (2004). Human MicroRNA Genes Are Frequently Located at Fragile Sites and Genomic Regions Involved in Cancers. Proc. Natl. Acad. Sci. USA.

[87] Wan L., Pantel K., Kang Y. (2013). Tumor Metastasis: Moving New Biological Insights into the Clinic. Nat. Med..

[88] Sun Y., Ma L. (2015). The Emerging Molecular Machinery and Therapeutic Targets of Metastasis. Trends Pharmacol. Sci..

[89] Kanchan R.K., Siddiqui J.A., Mahapatra S., Batra S.K., Nasser M.W. (2020). MicroRNAs Orchestrate Pathophysiology of Breast Cancer Brain Metastasis: Advances in Therapy. Mol. Cancer.

[90] Ma L. (2010). Role of MiR-10b in Breast Cancer Metastasis. Breast Cancer Res..

[91] Burk U., Schubert J., Wellner U., Schmalhofer O., Vincan E., Spaderna S., Brabletz T. (2008). A Reciprocal Repression between ZEB1 and Members of the MiR-200 Family Promotes EMT and Invasion in Cancer Cells. EMBO Rep..

[92] Brabletz S., Brabletz T. (2010). The ZEB/MiR-200 Feedback Loop—A Motor of Cellular Plasticity in Development and Cancer?. EMBO Rep..

[93] Kundu S.T., Byers L.A., Peng D.H., Roybal J.D., Diao L., Wang J., Tong P., Creighton C.J., Gibbons D.L. (2016). The MiR-200 Family and the MiR-183~96~182 Cluster Target Foxf2 to Inhibit Invasion and Metastasis in Lung Cancers. Oncogene.

[94] Ding X., Park S.I., McCauley L.K., Wang C.-Y. (2013). Signaling between Transforming Growth Factor β (TGF-β) and Transcription Factor SNAI2 Represses Expression of MicroRNA MiR-203 to Promote Epithelial-Mesenchymal Transition and Tumor Metastasis. J. Biol. Chem..

[95] Yu S.-J., Hu J.-Y., Kuang X.-Y., Luo J.-M., Hou Y.-F., Di G.-H., Wu J., Shen Z.-Z., Song H.-Y., Shao Z.-M. (2013). MicroRNA-200a Promotes Anoikis Resistance and Metastasis by Targeting YAP1 in Human Breast Cancer. Clin. Cancer Res..

[96] Mansoori B., Mohammadi A., Ghasabi M., Shirjang S., Dehghan R., Montazeri V., Holmskov U., Kazemi T., Duijf P., Gjerstorff M. (2019). MiR-142-3p as Tumor Suppressor MiRNA in the Regulation of Tumorigenicity, Invasion and Migration of Human Breast Cancer by Targeting Bach-1 Expression. J. Cell. Physiol..

[97] Li H., Rokavec M., Jiang L., Horst D., Hermeking H. (2017). Antagonistic Effects of P53 and HIF1A on MicroRNA-34a Regulation of PPP1R11 and STAT3 and Hypoxia-Induced Epithelial to Mesenchymal Transition in Colorectal Cancer Cells. Gastroenterology.

[98] Pencheva N., Tran H., Buss C., Huh D., Drobnjak M., Busam K., Tavazoie S.F. (2012). Convergent Multi-MiRNA Targeting of ApoE Drives LRP1/LRP8-Dependent Melanoma Metastasis and Angiogenesis. Cell.

[99] Wang S., Li W., Wen C., Diao Y., Zhao T. (2020). MicroRNA-214 Promotes the EMT Process in Melanoma by Downregulating CADM1 Expression. Mol. Med. Rep..

[100] Penna E., Orso F., Cimino D., Tenaglia E., Lembo A., Quaglino E., Poliseno L., Haimovic A., Osella-Abate S., De Pittà C. (2011). MicroRNA-214 Contributes to Melanoma Tumour Progression through Suppression of TFAP2C: MiR-214 and Melanoma Progression. EMBO J..

[101] Cantini L., Bertoli G., Cava C., Dubois T., Zinovyev A., Caselle M., Castiglioni I., Barillot E., Martignetti L. (2019). Identification of MicroRNA Clusters Cooperatively Acting on Epithelial to Mesenchymal Transition in Triple Negative Breast Cancer. Nucleic Acids Res..

[102] Lv Z.-D., Yang D.-X., Liu X.-P., Jin L.-Y., Wang X.-G., Yang Z.-C., Liu D., Zhao J.-J., Kong B., Li F.-N. (2017). MiR-212-5p Suppresses the Epithelial-Mesenchymal Transition in Triple-Negative Breast Cancer by Targeting Prrx2. Cell. Physiol. Biochem..

[103] Zhao L., Zhao Y., He Y., Mao Y. (2017). MiR-19b Promotes Breast Cancer Metastasis through Targeting MYLIP and Its Related Cell Adhesion Molecules. Oncotarget.

[104] Zhang L., Sullivan P.S., Goodman J.C., Gunaratne P.H., Marchetti D. (2011). MicroRNA-1258 Suppresses Breast Cancer Brain Metastasis by Targeting Heparanase. Cancer Res..

[105] Zhang L., Dong Y., Zhu N., Tsoi H., Zhao Z., Wu C.W., Wang K., Zheng S., Ng S.S., Chan F.K. (2014). MicroRNA-139-5p Exerts Tumor Suppressor Function by Targeting NOTCH1 in Colorectal Cancer. Mol. Cancer.

[106] Fan L., Wu Y., Wang J., He J., Han X. (2019). Sevoflurane Inhibits the Migration and Invasion of Colorectal Cancer Cells through Regulating ERK/MMP-9 Pathway by up-Regulating MiR-203. Eur. J. Pharmacol..

[107] Cai H., Chen X., Tang Y., Deng Y. (2017). MicroRNA-194 Modulates Epithelial–Mesenchymal Transition in Human Colorectal Cancer Metastasis. Onco Targets Ther..

[108] Martello G., Rosato A., Ferrari F., Manfrin A., Cordenonsi M., Dupont S., Enzo E., Guzzardo V., Rondina M., Spruce T. (2010). A MicroRNA Targeting Dicer for Metastasis Control. Cell.

[109] Shao Y., Chen T., Zheng X., Yang S., Xu K., Chen X., Xu F., Wang L., Shen Y., Wang T. (2018). Colorectal Cancer-Derived Small Extracellular Vesicles Establish an Inflammatory Premetastatic Niche in Liver Metastasis. Carcinogenesis.

[110] Wang D., Wang X., Si M., Yang J., Sun S., Wu H., Cui S., Qu X., Yu X. (2020). Exosome-Encapsulated MiRNAs Contribute to CXCL12/CXCR4-Induced Liver Metastasis of Colorectal Cancer by Enhancing M2 Polarization of Macrophages. Cancer Lett..

[111] Yang M., Chen J., Su F., Yu B., Su F., Lin L., Liu Y., Huang J.-D., Song E. (2011). Microvesicles Secreted by Macrophages Shuttle Invasion-Potentiating MicroRNAs into Breast Cancer Cells. Mol. Cancer.

[112] Zhou W., Fong M.Y., Min Y., Somlo G., Liu L., Palomares M.R., Yu Y., Chow A., O’Connor S.T.F., Chin A.R. (2014). Cancer-Secreted MiR-105 Destroys Vascular Endothelial Barriers to Promote Metastasis. Cancer Cell.

[113] Siegel R., Ward E., Brawley O., Jemal A. (2011). Cancer Statistics, 2011: The Impact of Eliminating Socioeconomic and Racial Disparities on Premature Cancer Deaths. CA. Cancer J. Clin..

[114] Siegel R.L., Miller K.D., Jemal A. (2016). Cancer Statistics, 2016: Cancer Statistics, 2016. CA. Cancer J. Clin..

[115] Mujoomdar A., Austin J.H.M., Malhotra R., Powell C.A., Pearson G.D.N., Shiau M.C., Raftopoulos H. (2007). Clinical Predictors of Metastatic Disease to the Brain from Non–Small Cell Lung Carcinoma: Primary Tumor Size, Cell Type, and Lymph Node Metastases. Radiology.

[116] Budczies J., von Winterfeld M., Klauschen F., Bockmayr M., Lennerz J.K., Denkert C., Wolf T., Warth A., Dietel M., Anagnostopoulos I. (2015). The Landscape of Metastatic Progression Patterns across Major Human Cancers. Oncotarget.

[117] Sørensen J.B., Hansen H.H., Hansen M., Dombernowsky P. (1988). Brain Metastases in Adenocarcinoma of the Lung: Frequency, Risk Groups, and Prognosis. J. Clin. Oncol..

[118] Zhu Z., Li Q., Xu M., Qi Z. (2020). Effect of Whole-Brain and Intensity-Modulated Radiotherapy on Serum Levels of MiR-21 and Prognosis for Lung Cancer Metastatic to the Brain. Med. Sci. Monit..

[119] Dong J., Zhang Z., Gu T., Xu S.-F., Dong L.-X., Li X., Fu B.-H., Fu Z.-Z. (2016). The Role of MicroRNA-21 in Predicting Brain Metastases from Non-Small Cell Lung Cancer. Onco Targets Ther..

[120] Singh M., Garg N., Venugopal C., Hallett R., Tokar T., McFarlane N., Mahendram S., Bakhshinyan D., Manoranjan B., Vora P. (2015). STAT3 Pathway Regulates Lung-Derived Brain Metastasis Initiating Cell Capacity through MiR-21 Activation. Oncotarget.

[121] Subramani A., Alsidawi S., Jagannathan S., Sumita K., Sasaki A.T., Aronow B., Warnick R.E., Lawler S., Driscoll J.J. (2013). The Brain Microenvironment Negatively Regulates MiRNA-768-3p to Promote K-Ras Expression and Lung Cancer Metastasis. Sci. Rep..

[122] Choi K.H., Shin C.H., Lee W.J., Ji H., Kim H.H. (2019). Dual-Strand Tumor Suppressor MiR-193b-3p and -5p Inhibit Malignant Phenotypes of Lung Cancer by Suppressing Their Common Targets. Biosci. Rep..

[123] Jiang C., Zhao H., Yang B., Sun Z., Li X., Hu X. (2020). Lnc-REG3G-3-1/MiR-215-3p Promotes Brain Metastasis of Lung Adenocarcinoma by Regulating Leptin and SLC2A5. Front. Oncol..

[124] Jiang W., Hou L., Wei J., Du Y., Zhao Y., Deng X., Lin X. (2021). Hsa-MiR-217 Inhibits the Proliferation, Migration, and Invasion in Non-Small Cell Lung Cancer Cells Via Targeting SIRT1 and P53/KAI1 Signaling. Balk. Med. J..

[125] Donzelli S., Mori F., Bellissimo T., Sacconi A., Casini B., Frixa T., Roscilli G., Aurisicchio L., Facciolo F., Pompili A. (2015). Epigenetic Silencing of MiR-145-5p Contributes to Brain Metastasis. Oncotarget.

[126] Zhao C., Xu Y., Zhang Y., Tan W., Xue J., Yang Z., Zhang Y., Lu Y., Hu X. (2013). Downregulation of MiR-145 Contributes to Lung Adenocarcinoma Cell Growth to Form Brain Metastases. Oncol. Rep..

[127] Hwang S.J., Lee H.W., Kim H.R., Song H.J., Lee D.H., Lee H., Shin C.H., Joung J.-G., Kim D.-H., Joo K.M. (2015). Overexpression of MicroRNA-95-3p Suppresses Brain Metastasis of Lung Adenocarcinoma through Downregulation of Cyclin D1. Oncotarget.

[128] Chen L., Xu S., Xu H., Zhang J., Ning J., Wang S. (2012). MicroRNA-378 Is Associated with Non-Small Cell Lung Cancer Brain Metastasis by Promoting Cell Migration, Invasion and Tumor Angiogenesis. Med. Oncol..

[129] Arora S., Ranade A.R., Tran N.L., Nasser S., Sridhar S., Korn R.L., Ross J.T.D., Dhruv H., Foss K.M., Sibenaller Z. (2011). MicroRNA-328 Is Associated with (Non-Small) Cell Lung Cancer (NSCLC) Brain Metastasis and Mediates NSCLC Migration. Int. J. Cancer.

[130] Wang H., Deng Q., Lv Z., Ling Y., Hou X., Chen Z., Dinglin X., Ma S., Li D., Wu Y. (2019). N6-Methyladenosine Induced MiR-143-3p Promotes the Brain Metastasis of Lung Cancer via Regulation of VASH1. Mol. Cancer.

[131] Liu J.-K., Liu H.-F., Ding Y., Gao G.-D. (2018). Predictive Value of MicroRNA Let-7a Expression for Efficacy and Prognosis of Radiotherapy in Patients with Lung Cancer Brain Metastasis: A Case–Control Study. Medicine.

[132] Wei C., Zhang R., Cai Q., Gao X., Tong F., Dong J., Hu Y., Wu G., Dong X. (2019). MicroRNA-330-3p Promotes Brain Metastasis and Epithelial-Mesenchymal Transition via GRIA3 in Non-Small Cell Lung Cancer. Aging.

[133] Chen L., Li X., Zhao Y., Liu W., Wu H., Liu J., Mu X., Wu H. (2017). Down-Regulated MicroRNA-375 Expression as a Predictive Biomarker in Non-Small Cell Lung Cancer Brain Metastasis and Its Prognostic Significance. Pathol. Res. Pract..

[134] Wu D., Deng S., Li L., Liu T., Zhang T., Li J., Yu Y., Xu Y. (2021). TGF-Β1-Mediated Exosomal Lnc-MMP2-2 Increases Blood–Brain Barrier Permeability via the MiRNA-1207-5p/EPB41L5 Axis to Promote Non-Small Cell Lung Cancer Brain Metastasis. Cell Death Dis..

[135] Mouttet D., Laé M., Caly M., Gentien D., Carpentier S., Peyro-Saint-Paul H., Vincent-Salomon A., Rouzier R., Sigal-Zafrani B., Sastre-Garau X. (2016). Estrogen-Receptor, Progesterone-Receptor and HER2 Status Determination in Invasive Breast Cancer. Concordance between Immuno-Histochemistry and MapQuant™ Microarray Based Assay. PLoS ONE.

[136] Berman A.T., Thukral A.D., Hwang W.-T., Solin L.J., Vapiwala N. (2013). Incidence and Patterns of Distant Metastases for Patients With Early-Stage Breast Cancer After Breast Conservation Treatment. Clin. Breast Cancer.

[137] Saha A., Ghosh S., Roy C., Choudhury K., Chakrabarty B., Sarkar R. (2013). Demographic and Clinical Profile of Patients with Brain Metastases: A Retrospective Study. Asian J. Neurosurg..

[138] Wilhelm I., Molnár J., Fazakas C., Haskó J., Krizbai I. (2013). Role of the Blood-Brain Barrier in the Formation of Brain Metastases. Int. J. Mol. Sci..

[139] Kennecke H., Yerushalmi R., Woods R., Cheang M.C.U., Voduc D., Speers C.H., Nielsen T.O., Gelmon K. (2010). Metastatic Behavior of Breast Cancer Subtypes. J. Clin. Oncol..

[140] Sereno M., Haskó J., Molnár K., Medina S.J., Reisz Z., Malhó R., Videira M., Tiszlavicz L., Booth S.A., Wilhelm I. (2020). Downregulation of Circulating MiR 802-5p and MiR 194-5p and Upregulation of Brain MEF2C along Breast Cancer Brain Metastasization. Mol. Oncol..

[141] Figueira I., Godinho-Pereira J., Galego S., Maia J., Haskó J., Molnár K., Malhó R., Costa-Silva B., Wilhelm I., Krizbai I.A. (2021). MicroRNAs and Extracellular Vesicles as Distinctive Biomarkers of Precocious and Advanced Stages of Breast Cancer Brain Metastases Development. Int. J. Mol. Sci..

[142] Debeb B.G., Lacerda L., Anfossi S., Diagaradjane P., Chu K., Bambhroliya A., Huo L., Wei C., Larson R.A., Wolfe A.R. (2016). MiR-141-Mediated Regulation of Brain Metastasis From Breast Cancer. J. Natl. Cancer Inst..

[143] Okuda H., Xing F., Pandey P.R., Sharma S., Watabe M., Pai S.K., Mo Y.-Y., Iiizumi-Gairani M., Hirota S., Liu Y. (2013). MiR-7 Suppresses Brain Metastasis of Breast Cancer Stem-Like Cells By Modulating KLF4. Cancer Res..

[144] Hwang S.J., Seol H.J., Park Y.M., Kim K.H., Gorospe M., Nam D.-H., Kim H.H. (2012). MicroRNA-146a Suppresses Metastatic Activity in Brain Metastasis. Mol. Cells.

[145] Xing F., Sharma S., Liu Y., Mo Y.-Y., Wu K., Zhang Y.-Y., Pochampally R., Martinez L.A., Lo H.-W., Watabe K. (2015). MiR-509 Suppresses Brain Metastasis of Breast Cancer Cells by Modulating RhoC and TNF-α. Oncogene.

[146] Pan J.-K., Lin C.-H., Kuo Y.-L., Ger L.-P., Cheng H.-C., Yao Y.-C., Hsiao M., Lu P.-J. (2021). MiR-211 Determines Brain Metastasis Specificity through SOX11/NGN2 Axis in Triple-Negative Breast Cancer. Oncogene.

[147] Tominaga N., Kosaka N., Ono M., Katsuda T., Yoshioka Y., Tamura K., Lötvall J., Nakagama H., Ochiya T. (2015). Brain Metastatic Cancer Cells Release MicroRNA-181c-Containing Extracellular Vesicles Capable of Destructing Blood–Brain Barrier. Nat. Commun..

[148] Xing F., Liu Y., Wu S.-Y., Wu K., Sharma S., Mo Y.-Y., Feng J., Sanders S., Jin G., Singh R. (2018). Loss of XIST in Breast Cancer Activates MSN-c-Met and Reprograms Microglia via Exosomal MiRNA to Promote Brain Metastasis. Cancer Res..

[149] Fong M.Y., Zhou W., Liu L., Alontaga A.Y., Chandra M., Ashby J., Chow A., O’Connor S.T.F., Li S., Chin A.R. (2015). Breast-Cancer-Secreted MiR-122 Reprograms Glucose Metabolism in Premetastatic Niche to Promote Metastasis. Nat. Cell Biol..

[150] Abbas O., Miller D.D., Bhawan J. (2014). Cutaneous Malignant Melanoma: Update on Diagnostic and Prognostic Biomarkers. Am. J. Dermatopathol..

[151] Knoll S., Fürst K., Kowtharapu B., Schmitz U., Marquardt S., Wolkenhauer O., Martin H., Pützer B.M. (2014). E2F1 Induces MiR-224/452 Expression to Drive EMT through TXNIP Downregulation. EMBO Rep..

[152] Rang Z., Yang G., Wang Y., Cui F. (2016). MiR-542-3p Suppresses Invasion and Metastasis by Targeting the Proto-Oncogene Serine/Threonine Protein Kinase, PIM1, in Melanoma. Biochem. Biophys. Res. Commun..

[153] Mikkelsen L.H., Andersen M.K., Andreasen S., Larsen A.-C., Tan Q., Toft P.B., Wadt K., Heegaard S. (2019). Global MicroRNA Profiling of Metastatic Conjunctival Melanoma. Melanoma Res..

[154] Bustos M.A., Tran K.D., Rahimzadeh N., Gross R., Lin S.Y., Shoji Y., Murakami T., Boley C.L., Tran L.T., Cole H. (2020). Integrated Assessment of Circulating Cell-Free MicroRNA Signatures in Plasma of Patients with Melanoma Brain Metastasis. Cancers.

[155] Yang X., Zhao H., Yang J., Ma Y., Liu Z., Li C., Wang T., Yan Z., Du N. (2019). MiR-150-5p Regulates Melanoma Proliferation, Invasion and Metastasis via SIX1-Mediated Warburg Effect. Biochem. Biophys. Res. Commun..

[156] Hanniford D., Zhong J., Koetz L., Gaziel-Sovran A., Lackaye D.J., Shang S., Pavlick A., Shapiro R., Berman R., Darvishian F. (2015). A MiRNA-Based Signature Detected in Primary Melanoma Tissue Predicts Development of Brain Metastasis. Clin. Cancer Res..

[157] Tan W.-S., Ho K.-S., Eu K.-W. (2009). Brain Metastases in Colorectal Cancers. World J. Surg..

[158] Li Z., Gu X., Fang Y., Xiang J., Chen Z. (2012). MicroRNA Expression Profiles in Human Colorectal Cancers with Brain Metastases. Oncol. Lett..

[159] Lee S.-J., Kim S.-J., Seo H.-H., Shin S.-P., Kim D., Park C.-S., Kim K.-T., Kim Y.-H., Jeong J.-S., Kim I.-H. (2012). Over-Expression of MiR-145 Enhances the Effectiveness of HSVtk Gene Therapy for Malignant Glioma. Cancer Lett..

[160] Wang J., Li B., Wang C., Luo Y., Zhao M., Chen P. (2019). Long Noncoding RNA FOXD2-AS1 Promotes Glioma Cell Cycle Progression and Proliferation through the FOXD2-AS1/MiR-31/CDK1 Pathway. J. Cell. Biochem..

[161] Kim C.W., Oh E.-T., Kim J.M., Park J.-S., Lee D.H., Lee J.-S., Kim K.K., Park H.J. (2019). Corrigendum to “Hypoxia-Induced MicroRNA-590-5p Promotes Colorectal Cancer Progression by Modulating Matrix Metalloproteinase Activity” [Cancer Lett. 416 (2018) 31–41]. Cancer Lett..

[162] Ljungberg B., Albiges L., Abu-Ghanem Y., Bensalah K., Dabestani S., Fernández-Pello S., Giles R.H., Hofmann F., Hora M., Kuczyk M.A. (2019). European Association of Urology Guidelines on Renal Cell Carcinoma: The 2019 Update. Eur. Urol..

[163] Jonasch E., Gao J., Rathmell W.K. (2014). Renal Cell Carcinoma. BMJ.

[164] Liu Y., Qi L., Zhang K., Wang F. (2021). MicroRNA-10a Suppresses Cell Metastasis by Targeting BDNF and Predicted Patients Survival in Renal Cell Carcinoma. J. BUON Off. J. Balk. Union Oncol..

[165] Heinzelmann J., Unrein A., Wickmann U., Baumgart S., Stapf M., Szendroi A., Grimm M.-O., Gajda M.R., Wunderlich H., Junker K. (2014). MicroRNAs with Prognostic Potential for Metastasis in Clear Cell Renal Cell Carcinoma: A Comparison of Primary Tumors and Distant Metastases. Ann. Surg. Oncol..

[166] Cai Y., Li H., Zhang Y. (2016). Downregulation of MicroRNA-206 Suppresses Clear Cell Renal Carcinoma Proliferation and Invasion by Targeting Vascular Endothelial Growth Factor A. Oncol. Rep..

[167] Song H., Rao Y., Zhang G., Kong X. (2018). MicroRNA-384 Inhibits the Growth and Invasion of Renal Cell Carcinoma Cells by Targeting Astrocyte Elevated Gene 1. Oncol. Res. Featur. Preclin. Clin. Cancer Ther..

[168] Dong J.-S., Wu B., Zha Z.-L. (2019). MicroRNA-588 Regulates Migration Capacity and Invasiveness of Renal Cancer Cells by Targeting EIF5A2. Eur. Rev. Med. Pharmacol. Sci..

